# Employer-provided bicycle benefit and changes in commuting and overall physical activity: A quasi-experiment among Finnish municipal employees

**DOI:** 10.5271/sjweh.4314

**Published:** 2026-09-01

**Authors:** Anna Makkonen, Essi Kalliolahti, Emilia Suomalainen, Jarno Tuominen, Paula Salo, Jenni Ervasti

**Affiliations:** 1 Department of Psychology and Speech-Language Pathology, University of Turku, Turku, Finland.; 2 Finnish Institute of Occupational Health, Helsinki, Finland.; 3 Finnish Environment Institute, Helsinki, Finland.

**Keywords:** active commuting, behavior change, health equity, longitudinal cohort, quasi-experimental study

## Abstract

**Objective:**

This study aimed to evaluate whether an employer-provided bicycle benefit changes commuting behavior and overall physical activity in a municipal workforce.

**Methods:**

Surveys from the Finnish Public Sector study in 2022 (pre-intervention) and 2024 (post-intervention) were used. The preregistered primary analysis was an intention-to-treat analysis. Employees in a municipality offering a bicycle benefit were propensity score-matched 1:2 to controls from a municipality without the benefit. Additional analyses included a per-protocol analysis (ie, the adopters) and an analysis of non-adopters (ie, nudge impact). Changes (2024 versus 2022) in weekly commuting kilometers were analyzed with propensity score-adjusted Poisson generalized estimating equation models.

**Results:**

In the intention-to-treat analysis, commuting by bicycle increased in the intervention group [rate ratio (RR) for 2024 compared with 2022=1.16, 95% confidence interval (CI) 1.04–1.30] compared with controls (RR 0.93, 95% CI 0.80–1.09; P for group × time interaction 0.02), corresponding to a 2.4 km weekly increase. Car commuting decreased (RR 0.89, 95% CI 0.83–0.96 versus RR 1.00, 95% CI 0.94–1.07; P=0.02), corresponding to a 7.7 km/week reduction (≈236 km/year) and ≈26 kg reduction in CO_2_e per person annually. No changes were observed in winter commuting or overall physical activity. Effects were larger among participants with optimal health and work ability. Among adopters, summer cycling increased by 14.4 km/week (RR 1.64, 95% CI 1.35–2.00). Non-adopters showed no changes.

**Conclusions:**

Availability of bicycle benefit increased bicycle commuting in summer weather and reduced car commuting, but effects were concentrated among healthier employees and adopters, suggesting that complementary measures may be needed to achieve broader health and climate impacts.

Daily commuting is a key opportunity to improve population health and mitigate climate change. Substituting car trips with cycling can substantially reduce transport-related emissions while increasing physical activity (PA), and pedal-assist electric bicycles (e-bikes) can extend these benefits to longer or more demanding trips (1–3). Meta-analytic evidence indicates that even moderate increases in cycling yield significant cardiometabolic and mortality benefits (4, 5). Modelled estimates also suggest that broader adoption of e-cycling could reduce passenger transport CO_2_ emissions by 1–11% at the population level, depending on uptake and travel substitution patterns (2, 3, 6). Despite the well-documented environmental and health benefits, in Finland, about 70% of commutes are made by car, and among municipal employees, only 4–19% cycle to/from work, depending on the season (7). As municipalities set climate targets, scalable ways to increase cycling, reduce car use, and, consequently, affect overall PA are highly relevant.

To encourage healthy and sustainable commuting, financial incentives have been developed. The UK 'Cycle to Work' scheme enables employees to lease bicycles tax-free via salary sacrifice, and evaluations suggest that the scheme increases cycling among both existing and new cyclists, with 38–48% reporting increased cycling frequency (8). Germany's company-bicycle leasing program has expanded rapidly (9), and a recent Norwegian quasi-experimental study found increased cycling among subsidy recipients but little change among non-recipients (10). Collectively, these programs indicate growing policy interest but also highlight that effects may be concentrated among adopters, leaving population-level impacts uncertain.

Despite this growing interest, scientific evidence of the effectiveness of this type of bicycle scheme and subsidies remains limited. Peer-reviewed studies to date include a qualitative analysis of organizational and individual motives for bicycle leasing in Germany (9), and European trials indicating that subsidized e-bike access can increase active travel, particularly among inactive or previously car-dependent adults (1, 11–14) However, no prior study has evaluated an employer-provided bicycle benefit using an intention-to-treat (ITT) design that captures the broader impact of *offering* the benefit, regardless of individual uptake, nor has any assessed whether impacts differ across employee health and work ability subgroups. It is also unclear whether such incentives lead to increased overall PA during both commute and leisure time or substitute for other forms of PA.

The present study addresses these gaps. We examined whether offering an employer-provided bicycle benefit increased bicycle commuting, decreased car commuting, and increased overall PA among municipal employees. We first applied the ITT approach to estimate the municipality-level impact of offering the bicycle benefit across all eligible employees. ITT provided greater statistical power (15, 16) and also enabled us to capture potential indirect effects, as some employees might increase cycling even without adopting the benefit. Such indirect change could reflect a ‘nudge' (17), a subtle behavior change strategy arising from social comparison or workplace visibility of the benefit where behavior shifts through peer influence rather than direct financial benefit (17–19). Therefore, we additionally conducted separate analyses among non-adopters to assess whether any nudge-like effects were observable. To distinguish any such indirect effects from changes driven by actual use of the benefit, we also analyzed adopters separately. These complementary analyses were necessary because ITT estimates alone cannot determine whether observed increases in cycling are attributable to nudge mechanisms or behavioral changes among benefit users.

Finally, including tailpipe CO_2_-equivalent (CO_2_e) emission calculations alongside changes in car commuting allows us to quantify the real-world environmental significance of these behavioral shifts, complementing the health- and activity-related outcomes.

Our preregistered primary objective was to use ITT analysis to estimate whether offering a bicycle benefit changes commuting behavior and overall PA (20). Our hypotheses were: (i) a bicycle benefit is associated with a more optimal trend or change in commuting by bicycle; (ii) a bicycle benefit is associated with a more optimal trend or change in overall PA during both commute and leisure time; and (iii) employees with suboptimal self-rated health or lifestyle risk factors benefit more (in terms of the most optimal trend or change in commuting by bicycle and overall PA) from the bicycle benefit than employees with optimal self-rated health and no such risk factors.

If the first hypothesis is supported, we will further investigate whether a corresponding decrease in car use accompanies the observed increase in cycling and estimate annual changes in tailpipe CO_2_e emissions.

## Methods

We examined whether offering an employer-provided bicycle benefit increased bicycle commuting, decreased car commuting, and increased overall PA among municipal employees.

### Intervention

In 2021, Finland introduced an employer-provided bicycle benefit (still in use during the study period) (21). The benefit is comparable to schemes implemented in several European countries for instance Belgium, The Netherlands, Austria, Germany, the UK, and Ireland. Such schemes are generally intended to promote healthier and more sustainable commuting through employer-supported cycling incentives (8). In Finland the scheme was introduced as a government-promoted incentive for healthier and more sustainable commuting (21). The employer leases the bicycle for employee use, including recreation. The benefit, offered as part of or in addition to salary, is tax-free up to €1200 annually, with any surplus treated as taxable income (21). In the intervention municipality, the benefit was introduced in June 2023. The decision to adopt the benefit was prompted by an increasing number of employee inquiries. Following discussion in the cooperation committee, a proposal was prepared, approved by the head of administration, and subsequently implemented after a procurement process. The program was managed in collaboration with a company that administered bicycle benefits. The purchase price of the bicycle and the approved cycling equipment were deducted over time from the employee's gross salary (€100 per month). The bike can be electric or traditional. While we have no information about which was purchased with the benefit, in 2024, roughly 30% of our survey respondents reported using an e-bike, and 70% reported using a traditional bike for commuting. The eligibility criteria for the bicycle benefit were as follows: a permanent employment contract (after the probationary period) or a fixed-term contract that lasts for the duration of the lease. This ensured that the employment contract was long enough to align with the bicycle benefit's payment schedule.

### Study population

This quasi-experimental study was nested within the Finnish Public Sector (FPS) study (22) approved by the ethics committee of the Hospital District of Helsinki and Uusimaa (HUS/1210/2016). We used the FPS survey time points in 2022 (before the intervention) and 2024 (after the intervention).

**Figure 1:**
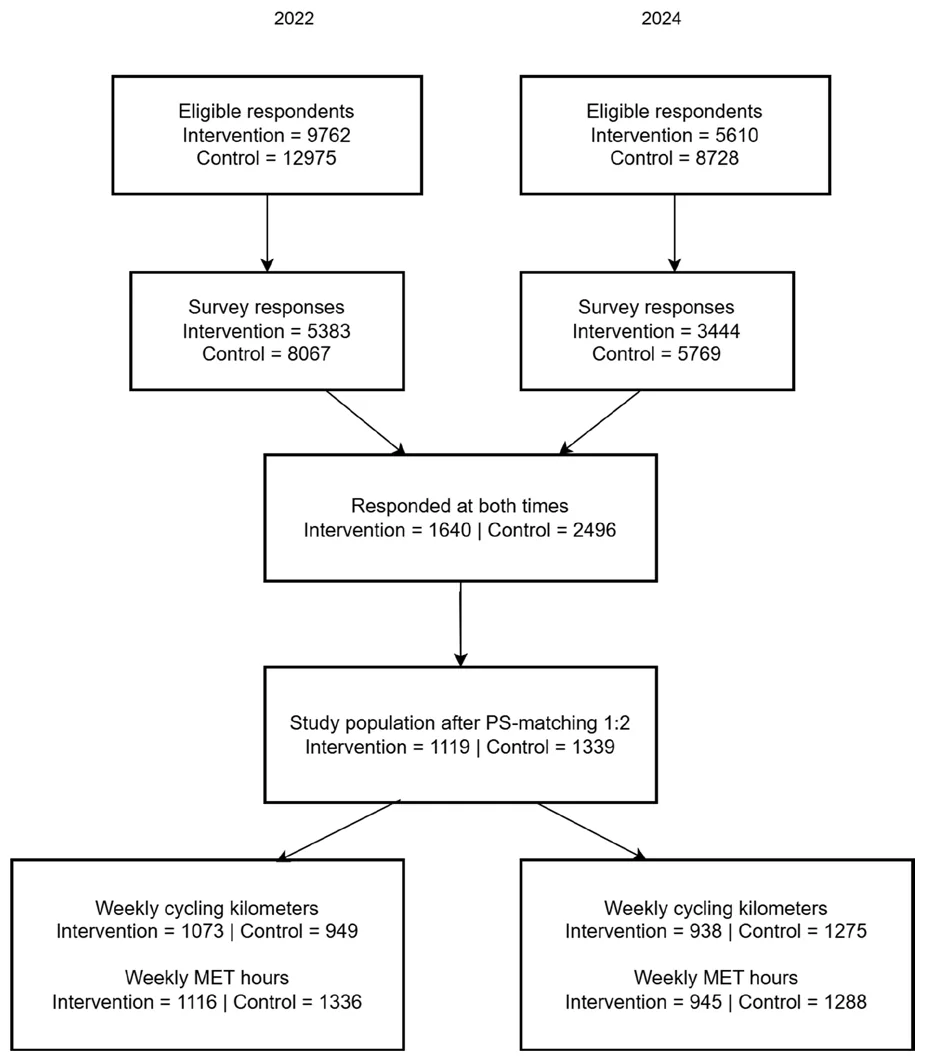
Flow chart of the study population in intention-to-treat analysis set.

In the ITT analyses, the intervention group consisted of employees of a Finnish municipality that offered a bicycle benefit. The control group consisted of employees of another municipality that did not provide such a benefit. In 2023, national health and social care reform transferred all health, social care, and rescue service employees to wellbeing counties, reducing the number of eligible respondents in 2024. A total of 1640 intervention participants and 2496 control participants completed both surveys and provided commuting data. The final analytic sample consisted of 1119 intervention participants and 1339 propensity score (PS)-matched controls from another municipality (figure 1).

For the per-protocol analysis of those taking the benefit (ie, the adopters) and non-adopter analysis, we used employer records on benefit recipients. Both analyses used PS matching at a 1:3 ratio with the same covariates as in the ITT analysis. The matched analytic samples included adopters (N=134 versus controls N=403) and non-adopters (N=997 versus controls N=1340) at both time points (2022 and 2024). These complementary analyses were necessary because ITT estimates alone cannot determine whether observed increases in cycling are attributable to nudge mechanisms or behavioral changes among benefit users.

Flow charts of the per-protocol and non-adopters are presented in the supplementary material (www.sjweh.fi/

article/4314), figures S1 and S2.

### Measures

Commuting modes (summer/winter) were assessed on a five-category frequency scale for walking, cycling, public transport, and private car use (23, 24). Weekly kilometers were derived from frequency × round-trip distance. No metabolic equivalent of task (MET) conversion or standard MET values were applied to the cycling commuting variables. Overall PA during both commute and leisure time was measured with a validated four-level question (past 12 months) and converted to MET-hours using standard values (25). Covariates included age, sex, socioeconomic status, contract type, job tenure, working time, remote work, marital status, children, and commuting distance, alcohol consumption (26), smoking (27), job strain (28–30), and recovery from work (31–34). Subgroup analyses were based on self-rated health (34), self-rated work ability (35), and body mass index (BMI) (<30 versus ≥30 kg/m^2^) (36). Full coding rules, operational definitions, imputations, and calculation formulas are provided in supplement 1.

Annual tailpipe CO_2_e was estimated from the summer weather reduction in weekly car-commuting kilometers, assuming eight summer (snow-free) months from April to November and 30.7 working weeks (37, 38) and an emission factor of 110 g/km (39). Total avoided emissions equal per-employee change × number of eligible employees, benchmarked against typical annual passenger-car emissions.

### Statistical analysis

The PS method was used to approximate comparability between intervention participants and controls. The PS is the conditional probability of being assigned ‘intervention', given the observed covariates, ie, confounders (40). We created the intervention and comparison groups using a logistic regression model predicting participation in the intervention, with baseline characteristics (age, sex, socioeconomic status, contract type, job tenure, working time, remote work, marital status, underage children, smoking, alcohol use, job strain, recovery from work, and commuting distance) as covariates. In the ITT analyses, each intervention case was matched 1:2 with controls with the same PS. We did not match cases and controls by work ability, BMI, or self-rated health, as these variables were used later for stratified analyses. Standardized mean differences (SMD) were used to assess group balance, including work ability, BMI, and self-rated health. SMD was also calculated for work ability, BMI, and self-rated health. The same procedure was used for the per-protocol and non-adopters analysis, but we used PS-matching at a 1:3 ratio. This was done to ensure sufficient statistical power, given the smaller groups in these analyses.

To determine changes in commuting by bicycle, by car, and overall PA from 2022 to 2024, we applied a repeated-measures Poisson regression analysis using generalized estimating equations (GEE) with an exchangeable correlation structure. This method accounts for the intraindividual correlation between measurements. We calculated the rate ratio (RR) and its 95% confidence interval (CI) by contrasting year 2024 with year 2022. Analyses were adjusted for PS. To analyze whether trends differed between 2024 and 2022 among intervention cases and controls, we tested the time × group interaction.

To demonstrate absolute levels of active commuting and, overall PA among cases and controls, we calculated the annual (2022, 2024) least-squares means for both groups. Additionally, as we noted an association between the intervention and summertime cycling, we used GEE to estimate changes in car commute from 2022 to 2024. Finally, including tailpipe CO_2_-equivalent (CO_2_e) emission calculations alongside changes in car commuting allows us to quantify the real-world environmental significance of these behavioral shifts, complementing the health- and activity-related outcomes.

The SAS software package version 9.4 (SAS Institute, Cary, NC, USA) was used for statistical analyses. Figures were created in R version 4.4.1 (R Foundation for Statistical Computing) using ggplot2 and patchwork packages.

## Results

Baseline characteristics for the intervention and control groups in the ITT analysis are shown in table 1. Women comprised 75–78% of the population, the mean age was 47.7 years, 95% worked daytime, and baseline characteristics were well balanced (SMD <10%). Baseline characteristics for the per-protocol and non-adopters samples were similarly well balanced after 1:3 PS matching (supplementary tables S1–S2).

**Table 1:** Baseline characteristics of propensity score-matched employees in intervention and control groups. Intention-to-treat analysis. [SMD=standardized mean difference; SD=standard deviation].

Baseline characteristics		Intervention (N=1119)			Control (N=1339)		SMD
		N (%)	Mean (SD)		N (%)	Mean (SD)	
**Categorical variables**							
Sex							
	Women	837 (74.8)			1040 (77.7)		7
	Men	282 (25.2)			299 (22.3)		
Socioeconomic status							
	High	882 (78.8)			1096 (81.9)		-6.4
	Intermediate	143 (12.8)			124 (9.3)		-2.8
	Low	94 (8.4)			119 (8.9)		-7.9
Working time							
	Day work	1073 (95.9)			1298 (96.9)		1.7
	Shift work	46 (4.1)			41 (3.1)		
Smoking							
	Yes	60 (5.4)			85 (6.4)		7.1
	No	1059 (94.6)			1254 (93.7)		
Alcohol use							
	No	178 (15.9)			275 (20.5)		-4.7
	Low-risk use	899 (80.3)			1022 (76.3)		-4.7
	High-risk use	42 (3.8)			42 (3.1)		2.7
Marital status							
	Single	126 (11.3)			147 (11.0)		-2.6
	Married/cohabiting	877 (78.4)			1039 (77.6)		2.7
	Divorced/widowed	116 (10.4)			153 (11.4)		2.3
Underaged children							
	Yes	350 (31.3)			424 (31.7)		-0.7
	No	769 (68.7)			915 (68.3)		
Job strain							
	Yes	140 (12.5)			177 (13.2)		-2.5
	No	979 (87.5)			1162 (86.8)		
**Continuous variables**							
Age, years			47.7 (9.24)			47.7 (9.89)	-7.3
Recovery from work			6.50 (2.22)			6.42 (2.34)	0.7
Work ability			8.04 (1.59)			8.11 (1.51)	3.7
Body mass index			26.57 (4.79)			26.30 (4.68)	-7
Self-rated health			4.13 (0.86)			4.17 (0.87)	

### Main results

**Figure 2:**
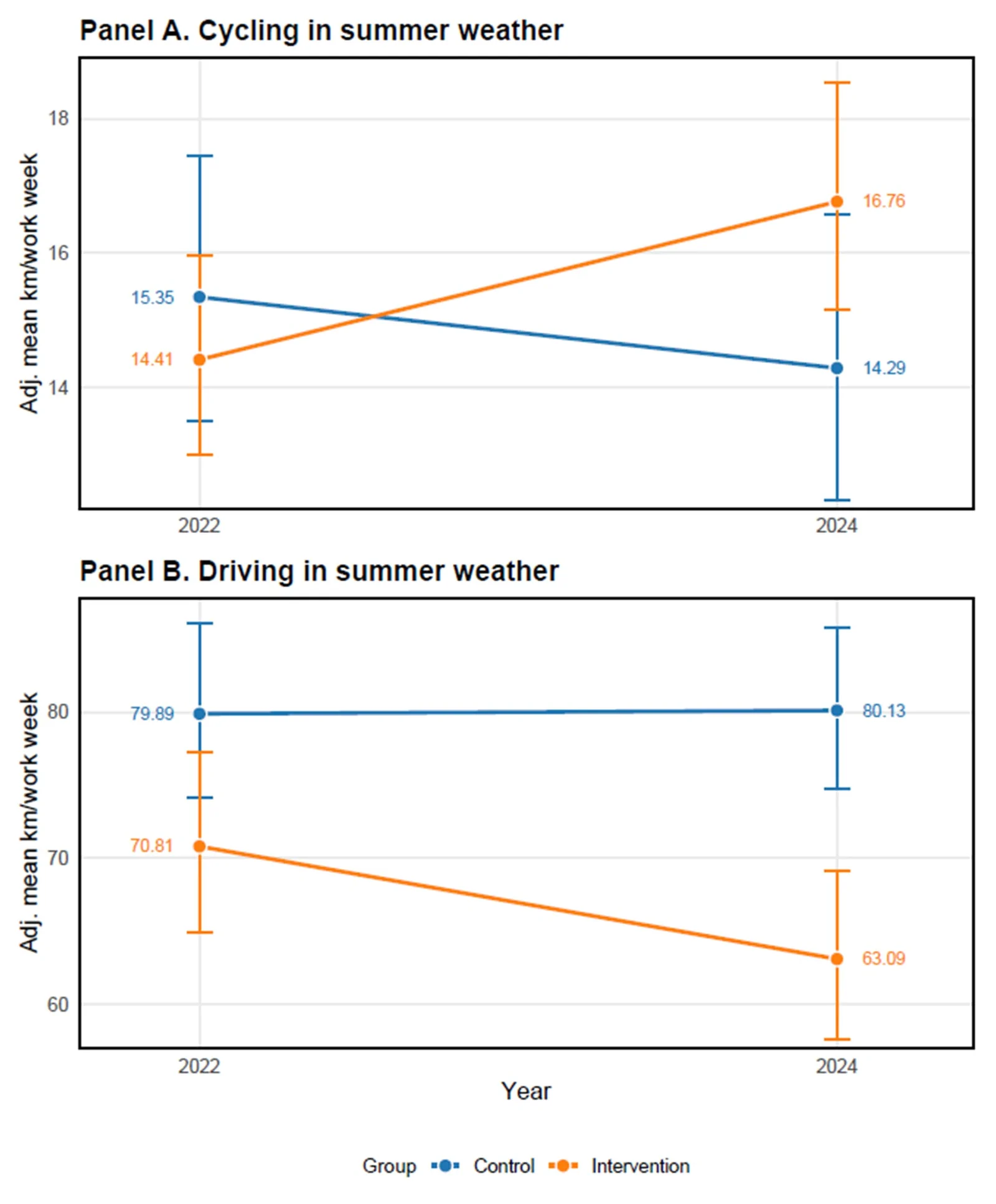
Adjusted means of weekly kilometers commuted by bicycle. Panel A: Cycling in summer weather. Panel B: Car commuting in summer weather. Error bars indicate 95% confidence intervals. The results shown are from the ITT analysis.

In the ITT analysis, summer weather bicycle commuting increased from 2022 to 2024 in the intervention group (RR_2024 vs 2022_=1.16, 95% CI 1.04–1.30), while no change was observed in controls (0.93, 0.80–1.09, time × group interaction P=0.02) (table 2). This corresponded to an increase of 2.4 km of bicycle commuting weekly (figure 2). No change was observed in winter weather bicycle commute (table 2, supplementary figure S4) or in overall PA (table 2). PS-adjusted least-squares means with 95% CI for each group and year are provided in supplementary table S3.

**Table 2:** Bicycle commute (weekly kilometers), overall weekly physical activity (MET hours per week), and car commute after the intervention versus before the intervention. [RR=rate ratio; CI=confidence intervals].

Analysis		Bicycle commute (summer weather)			Bicycle commute (winter weather)			Overall physical activity			Car commute (summer weather)	
		RR (95% CI)	P-value ^a^		RR (95% CI)	P-value ^a^		RR (95% CI)	P-value ^a^		RR (95% CI)	P-value ^a^
Intention-to-treat			0.02			0.53			0.71			0.02
	Intervention	1.16 (1.04–1.30)			1.08 (0.91–1.28)			1.03 (0.98–1.08)			0.89 (0.83–0.96)	
	Control	0.93 (0.80–1.09)			0.95 (0.66–1.38)			1.02 (0.97–1.06)			1.00 (0.94–1.07)	
Per protocol			0.004			0.41			0.74			0.17
	Intervention	1.64 (1.35–2.00)			1.56 (1.20–2.02)			1.08 (0.95–1.22)			0.80 (0.68–0.94)	
	Control	1.04 (0.82–1.31)			1.22 (0.73–2.03)			1.05 (0.94–1.16)			0.97 (0.78–1.21)	
Non-adopters			0.7						0.99			0.64
	Intervention	1.04 (0.92–1.17)			1.04 (0.85–1.28)			1.00 (0.95–1.05)				
	Control	1.00 (0.85–1.17)			1.05 (0.72–1.53)			1.02 (0.97–1.06)				

^a^ P for interaction.

The per-protocol analysis suggested even stronger associations between bicycle benefit and bicycle commuting. Among adopters, summer weather bicycle commuting increased (RR_2024 vs 2022_=1.64, CI 1.35–2.00), whereas no change was observed among controls (RR_2024 vs 2022_=1.04, 95% CI 0.82–1.31; P for time × group interaction=0.004). On average, this change in the intervention group corresponded to an additional 14.4 weekly kilometers cycled to work (supplementary figure S3). For winter weather bicycle commuting, the time × group interaction was non-significant, suggesting no difference between intervention and control participants (supplementary figure S4). Among non-adopters, no changes in cycling or overall PA were observed (table 2.)

### Subgroup analyses

In the ITT analysis among employees with optimal work ability, summer weather commuting by bicycle increased in the intervention group compared to controls (RR_2024 vs 2022_=1.18, CI 1.03–1.35; control RR_2024 vs 2022=_0.92, CI 0.76–1.12; time × group interaction P=0.04). In the suboptimal work ability subgroup, no such change was observed. In a similar vein, summer weather commuting by bicycle increased in the intervention group among employees with good self-rated health (RR_2024 vs 2022_=1.17, CI 1.03–1.33) but not among controls (RR_2024 vs 2022_=0.92, CI, 0.77–1.10; P=0.02). No change in bicycle commuting was observed among participants with suboptimal self-rated health in the intervention group. The subgroup analysis based on BMI showed no differences between intervention and control groups, neither in those with a healthy weight nor in those with BMI indicating obesity (table 3).

**Table 3:** Summer weather commute by bicycle (weekly km): rate ratios (RR, 2024 vs. 2022) from PS-adjusted generalized estimating equations (GEE) models by subgroups (defined at baseline) with 95% confidence intervals (CI). Models include only respondents with both 2022 and 2024 data for the outcome. [N obs=number of observations; BMI=body mass index; NA=not estimable (GEE Type III score test DF=0) due to too few repeated observations in the intervention subgroup ].

Analysis set	Subgroup	Intervention		Control		Intervention		Control	P for time×group
		N obs		N obs		RR (95% CI)		RR (95% CI)	
Intention-to-treat	Healthy weight	770		932		1.11 (0.94–1.31)		0.90 (0.74–1.09)	0.08
Intention-to-treat	Obesity (BMI ≥30)	1159		1202		1.29 (1.29–1.51)		1.02 (0.76–1.36)	0.16
Intention-to-treat	Optimal work ability	1506		1645		1.18 (1.03–1.35)		0.92 (0.76–1.12)	0.04
Intention-to-treat	Suboptimal work ability	504		576		0.95 (0.71–1.27)		1.28 (0.98–1.65)	NA
Intention-to-treat	Good health	1546		1745		1.17 (1.03–1.33)		0.92 (0.77–1.10)	0.02
Intention-to-treat	Suboptimal health	464		472		1.09 (0.82–1.43)		1.09 (0.75–1.60)	0.98
Per protocol	Healthy weight	44		136		1.69 (1.21–2.36)		1.11 (0.83–1.49)	0.05
Per protocol	Obesity (BMI ≥30)	49		135		1.53 (1.18–1.99)		1.22 (0.82–1.81)	NA
Per protocol	Optimal work ability	96		273		1.72 (1.37–2.16)		1.03 (0.79–1.35)	0.00
Per protocol	Suboptimal work ability	32		83		1.40 (0.92–2.11)		1.07 (0.62–1.86)	0.47
Per protocol	Good health	101		283		1.79 (1.44–2.24)		1.02 (0.79–1.32)	0.00
Per protocol	Suboptimal health	28		71		1.22 (0.72–2.06)		1.41 (0.74–2.67)	0.74

In the per-protocol analyses, summer weather commute by bicycle increased markedly among adopters with optimal work ability (RR_2024 vs 2022_=1.72, CI 1.37–2.16), with good self-rated health (RR_2024 vs 2022_= 1.79, CI 1.44–2.24), and with a healthy weight (RR_2024 vs 2022_=1.69 and 1.53) compared to controls (P values for group × time interactions were ≤0.05). The corresponding results among those with suboptimal work ability, suboptimal health, and obesity showed either no difference between intervention and control groups or were inestimable (table 3).

### Car commuting and emission reduction

As prespecified, because bicycle commuting in summer weather increased in the ITT and per-protocol analyses, we examined whether this shift was reflected also as a reduction in car commuting. In the ITT analysis, summer weather car commuting decreased in the intervention group (RR_2024 vs 2022_=0.89, 95% CI 0.83–0.96), whereas no change was observed in the control group (RR_2024 vs 2022_=1.00, 95% CI 0.94–1.07; P for time × group interaction=0.02). While we observed a similar decrease in per-protocol analysis, the time × group interaction term did not reach statistical significance, thus suggesting no difference between intervention and control groups (table 2).

The emission calculations were based on the ITT analysis. We assumed that all employees in the intervention municipality would experience the same average reduction in weekly summer weather car commuting of 7.7 km (figure 2). Importantly, this is not the reduction achieved by adopters alone, but the estimated per-employee effect for all those eligible to the bicycle benefit. Under this assumption, each employee would reduce their annual car commuting by approximately 236 km, corresponding to 26 kg of CO_2_e per person per year. When scaled to all 5610 eligible employees, the total reduction amounts to 146 tonnes of CO_2_e annually.

## Discussion

The preregistered ITT design, complemented by per-protocol and non-adopter analyses, distinguished population-level from individual effects. Offering the bicycle benefit led to a modest increase in summer bicycle commutes and a small but meaningful reduction in car commutes. Changes were more pronounced in healthier employees and those with better work ability. Non-adopters showed no change, whereas adopters increased bicycle commuting substantially.

Despite an increase of 2.4 commuted weekly kilometers by bicycle in summer weather, there was no change in commute modes in winter weather. Prior work shows that snow, ice, cold, and darkness reduce winter cycling in Finland and even in higher-cycling contexts such as Germany (5, 6, 41, 42). In Finland, cycling decreases sharply in winter, even in cities known for well-maintained cycle paths (5, 43). Among adopters, summer bicycle commuting increased by approximately 14 km/week. Comparisons with earlier interventions are somewhat limited because studies have reported cycling exposure using different measures. For example, Höchsmann et al (13) reported total commuting distance accumulated during an e-bike intervention, whereas de Kruijf et al (14) reported changes in the number of cycling trips and modal share rather than absolute weekly cycling kilometers, making direct quantitative comparison difficult. Meta-analytic evidence shows pooled increases of around 15–25 km/week among intervention participants (1). The observed weekly increase among adopters is therefore lower than in high-intensity interventions but well aligned with effects from self-directed or incentive-based programs that do not include free equipment or structured behavioral support. This pattern also mirrors the UK Cycle to Work evaluation, which reported increases among adopters but did not quantify weekly kilometers (8). The additional cycling (~40–50 min/week) represents a dose linked to 10–20% lower all-cause and cardiovascular mortality in meta-analytic evidence (4, 44, 45), underscoring its public health potential even when car use does not decline proportionately.

Although cycling increased in both ITT and per-protocol analyses, overall PA did not. Previous studies have reported mixed results on whether increased active commuting is associated with higher overall PA. Some have found clear gains in overall PA (46, 47), whereas others, such as Novis et al (48), observed no net change. Several mechanisms may explain our findings. Added cycling likely replaced other activities, such as leisure exercise or walking to public transport, rather than increasing total energy expenditure. Moreover, e-bikes, the majority of benefit bicycles, enable longer but less intense rides, increasing distance without proportional MET-hour gains (49). Finally, the use of a self-reported 12-month recall measure may have limited sensitivity to detect modest within-person changes (50). Together, these factors suggest that the absence of a measurable increase in overall PA does not necessarily imply unchanged energy expenditure but rather reflects partial substitution and methodological constraints.

The observed reduction of 7.7 km in weekly car commuting in our ITT analysis corresponds to roughly a 10–11% decrease in summer car-use distance among all eligible municipal employees and to an estimated 146 tCO_2_e reduction. Compared with modelled population-level scenarios, the observed reductions reflect the constrained scale of a targeted intervention. The observed reductions were much smaller than national modelled estimates predicting up to 56 kt CO_2_e savings over five years among working-age adults on commute trips (N=3409) (6). Consistently, Mesimäki et al (51) estimated that substituting all realistically cyclable car trips across all trip purposes would reduce national passenger car CO_2_ emissions by <1%. As expected, our municipality-level effect was smaller than participant-level reductions, since ITT includes many employees who did not change their behavior. For example, Chevance et al. found in their meta-analysis that behavioral active-travel interventions reduced car travel by 2.4 km/day and car mode share by about 10% among intervention participants, roughly twice the weekly reduction observed in our population-level estimate (1).

Comparable evidence from Nordic incentive schemes reinforces this interpretation. In Oslo's e-bike subsidy program, participants reduced their car travel by about 1.3 km per day after receiving a subsidy for e-bike purchase (10). Although the study focused on a cost–benefit analysis rather than emissions per se, its findings align closely with ours. The monetary value of CO_2_e reductions was negligible compared with the program's broader health-related and economic benefits.

Against this backdrop, the climate impact of the bicycle-benefit scheme appears modest yet consistent with what can be expected from a voluntary, employer-based measure. Compared with pricing or regulatory instruments, such as fuel taxes or congestion charging, that can reduce car-related emissions by 10–15% within targeted zones while generating public revenue (52–54), voluntary incentives typically achieve smaller per-person reductions but are generally more publicly acceptable.

Improvements were concentrated among employees with optimal work ability and better health, a pattern consistent with findings from the UK Cycle to Work scheme evaluation, which showed that uptake and cycling increases were concentrated among more advantaged employees (eg, higher-income and professional groups) rather than evenly across the workforce (8). This suggests that a mere offer may primarily benefit those already likely to cycle. Reaching groups with poorer baseline health or lower PA is particularly important, as the health benefits of increasing activity tend to be greatest among those who start from lower fitness or activity levels. Extensive cohort studies show that even modest increases in walking or cycling are associated with disproportionately larger reductions in cardiometabolic risk and mortality among previously inactive or higher-risk individuals (5, 55, 56). Evidence from controlled e-bike trials further indicates that electrically assisted cycling can elicit clinically meaningful improvements in fitness among overweight or previously inactive adults (13). Moreover, recent implementation studies, such as the Milwaukee bicycling intervention among lower-income inactive adults (57) and the Australian behavioral-support e-bike program (58), demonstrate that tailored, low-threshold enabling measures can successfully engage higher-risk groups. Taken together, structural incentives like the bicycle benefit may need to be complemented with additional support to reach those who could gain the most.

Employees who did not adopt the bicycle benefit showed no increase in cycling or decrease in car commuting. Thus, we found no evidence that the benefit operated as a nudge, increasing cycling among non-adopters. Prior nudge-based interventions have shown, at best, modest, context-dependent effects and usually require more direct targeting of individuals (59). Nevertheless, a light voluntary bicycle benefit could still plausibly inspire colleagues to cycle through increased social visibility and the gradual emergence of workplace norms, effects that may unfold over longer time horizons than the two-year observation period considered here. The comparison between adopters and non-adopters in our data still parallels the three-group structure used by Veisten et al., who found that eligible non-recipients (used as a control group), exhibited no behavioral change despite exposure to the same e-bike subsidy offer (10). To our knowledge, this represents the only previous study to examine whether such a benefit could influence the commuting behavior of non-adopters, making the present study the first to test this indirect mechanism explicitly within a bicycle benefit scheme.

### Strengths and limitations

Key strengths of this study include the use of three complementary analytic sets (ITT, per-protocol, non-adopters) and PS-matched DiD–GEE models, improving interpretability and policy relevance. A further strength is that cycling behavior was quantified in kilometers and days. The real-world policy context enhances external validity.

However, several limitations should be acknowledged. Statistical power was lower in the subgroup and per-protocol analyses due to smaller sample sizes, possibly limiting the detection of modest effects. With only two timepoints over two years, the longer-term sustainability of behavioral change could not be assessed. The framework of studying difference in differences assumes parallel trends (60), but with only one intervention and one control municipality, deviations in pre-trends or unobserved contextual factors (eg, new infrastructure, parking policies, fuel prices, or workplace initiatives) cannot be ruled out. Furthermore, the study was conducted in two large Finnish cities, which may limit generalizability to more rural areas, where longer commuting distances and fewer alternatives to the private car may enable e-bikes to achieve greater reductions in driving, as suggested by recent meta-analytic evidence (1).

Moreover, all outcomes, including commuting frequency, kilometers, and overall PA were self-reported, which may introduce measurement error and either deflate or attenuate effect estimates. In addition, we lacked detailed data on bicycle type among benefit adopters, which prevented subgroup analyses comparing electric and traditional bicycle users, despite potential differences in PA intensity and energy expenditure between these modes (49). Objective measures (eg, GPS) could provide more accurate behavioral data in future studies. Nevertheless, self-reported commuting mode is generally recalled with acceptable group-level validity and reliability in surveys and travel diaries (61, 62).

Selection bias is also possible even after statistical adjustment if employees in the intervention municipality were more health- or environmentally oriented at baseline. Our sample was primarily women (≈75%), reflecting the Finnish municipal workforce and potentially limiting generalizability to male-dominated sectors.

Finally, in the ITT analyses, car-use distance decreased more than cycling increased, indicating that part of the estimated emission reduction may not be directly attributable to the intervention. Thus, the intervention-attributable emission reduction may be overestimated, even if the total reduction in emissions is not.

### Concluding remarks

The findings suggest that the bicycle benefit promotes summer weather active commuting and may thus yield meaningful public health benefits, though its impact on emissions was small. The policy has also stimulated business activity in the cycling sector, yet its advantages are unevenly distributed: higher-income and healthier employees were most likely to adopt the benefit, while lower-wage or temporary workers often remained excluded. Despite the widespread use of e-bikes among beneficiaries, our results did not indicate an increase in winter cycling, nor did the benefit appear to engage employees with poorer health or lower fitness. We also found no evidence of indirect nudge effects among non-adopters. In addition, leasing multiple or high-end bicycles may offset environmental gains through increased material consumption. Although the per-person impact is modest, the cumulative potential could become significant if similar schemes were implemented widely across employers or municipalities and better targeted to lower-income individuals and those who would benefit most from increased PA.

## Supplementary material

Supplementary material
