# Supplementary material for: Employer-provided bicycle benefit and changes in commuting and overall physical activity: A quasi-experiment among Finnish municipal employees
Source: Scand J Work Environ Health. 2026 Aug 31;52(5):582–92. doi: 10.5271/sjweh.4314 (PMC13551496; doi:10.5271/sjweh.4314)
Supplement: Supplementary file 1 [file SJWEH-52-582-S001.pdf]

Employer-provided bicycle benefit and changes in commuting and overall physical activity: A quasi-experiment among Finnish municipal employees[1]

by Anna Makkonen, MA,*[2] *Essi Kalliolahti, PhD, Emilia Suomalainen, PhD, Jarno Tuominen, PhD, Paula Salo, PhD, Jenni Ervasti, PhD

1. SUPPLEMENTARY MATERIAL
2. Correspondence to: Anna Makkonen, Faculty of Psychology and Speech-Language Pathology, 20014 Turku, Finland. [Email: anna.makkonen@utu.fi]

Supplement 1. Supplemental tables and figures.

Table S1. Baseline characteristics of propensity score-matched employees in intervention and control groups. Analysis set: Per protocol. Standardized mean difference (SMD) values below 10% indicate a good balance between groups. SD=standard deviation.

Baseline characteristic	Intervention (N=134) N (%)	Control (N=403) N (%)	SMD (%)
**Sex**:			
Women	93 (69.4)	283 (70.2)	-4.2
Men	41 (30.6)	120 (29.8)	
**Mean age** (SD)	48.0(8.3)	48.7 (9.2)	0.6
**Socioeconomic status**:			
High	116 (86.6)	347 (86.1)	1.1
Intermediate	8 (6.0)	25 (6.2)	4.3
Low	10 (7.5)	31 (7.7)	6.3
**Working time**:			
day work	127 (94.8)	382 (94.8)	4.1
Shift work	7 (5.2)	21 (5.2)	

**Smoking**:			
Yes	2 (1.5)	13 (3.2)	-3.5
No	132 (98.5)	390 (96.8)	
**Alcohol use**:			
No	17 (12.7)	39 (9.7)	4.6
Low risk use	112 (83.6)	344 (85.4)	-2.8
High risk use	5 (3.7)	20 (5.0)	0.7
**Marital status**:			
Single	12 (9.0)	29 (7.2)	-0.8
Married/cohabiting	108 (80.6)	334 (82.9)	0.7
Divorced/widowed	14 (10.4)	40 (9.9)	-3.0
**Underaged children**:			
Yes	44 (32.8)	123 (30.5)	5.0
No	90 (67.2)	280 (69.5)	
**Job strain**:			
Yes	14 (10.4)	40 (9.9)	3.5
No	120 (89.6)	363 (90.1)	
**Mean age** (SD)	48.04 (8.34)	48.68 (9.19)	0.6
**Mean work ability** (SD)	8.18 (1.36)	8.11 (1.54)	-2.4
**Mean body mass index** (SD)	26.92 (4.63)	26.29 (4.62)	-11.4
**Mean recovery from work** (SD)	6.69 (2.07)	6.64 (2.21)	-1.1
**Mean self-rated health** (SD)	4.15 (0.84)	4.18 (0.83)	0.4

Table S2. Baseline characteristics of propensity score-matched employees in intervention and control groups. Analysis set: Non-adopters. Standardized mean difference (SMD) values below 10% indicate a good balance between groups. SD=standard deviation.

**Baseline characteristic**	**Intervention (N=997) N (%)**	**Control (N=1340) N (%)**	**SMD (%)**
**Sex**:			
Women	746 (74.8)	1051 (78.4)	10.9
Men	251 (25.2)	289 (21.6)	
Mean age (SD)	48.2(9.1)	47.6(9.8)	-9.6
**Socioeconomic status**:			
High	782 (78.4)	1093 (81.6)	-6.6
Intermediate	129 (12.9)	135 (10.1)	-8.1
Low	86 (8.6)	112 (8.4)	-8.8
**Working time**:			
day work	947 (95.0)	1295 (96.6)	0.9
Shift work	50 (5.0)	45 (3.4)	
**Smoking**:			
Yes	54 (5.4)	85 (6.3)	6.4
No	943 (94.6)	1255 (93.7)	
**Alcohol use**:			
No	170 (17.1)	273 (20.4)	-3.9
Low risk use	782 (78.4)	1025 (76.5)	-5.0
High risk use	45 (4.5)	42 (3.1)	0.7
**Marital status**:			

Single	114 (11.4)	157 (11.7)	0.1
Married/cohabiting	775 (77.7)	1018 (76.0)	0.7
Divorced/widowed	108 (10.8)	165 (12.3)	4.8
**Underaged children**:			
Yes	306 (30.7)	436 (32.5)	0.2
No	691 (69.3)	904 (67.5)	
**Job strain**:			
Yes	125 (12.5)	181 (13.5)	-6.0
No	872 (87.5)	1159 (86.5)	
**Mean age** (SD)	48.15 (9.10)	47.61 (9.79)	-9.6
**Mean work ability** (SD)	8.02 (1.61)	8.09 (1.53)	6.2
**Mean body mass index** (SD)	26.54 (4.83)	26.29 (4.74)	-7.3
**Mean recovery from work** (SD)	6.54 (2.15)	6.41 (2.37)	-0.6
**Mean self-rated health** (SD)	4.11 (0.86)	4.16 (0.88)	8.1

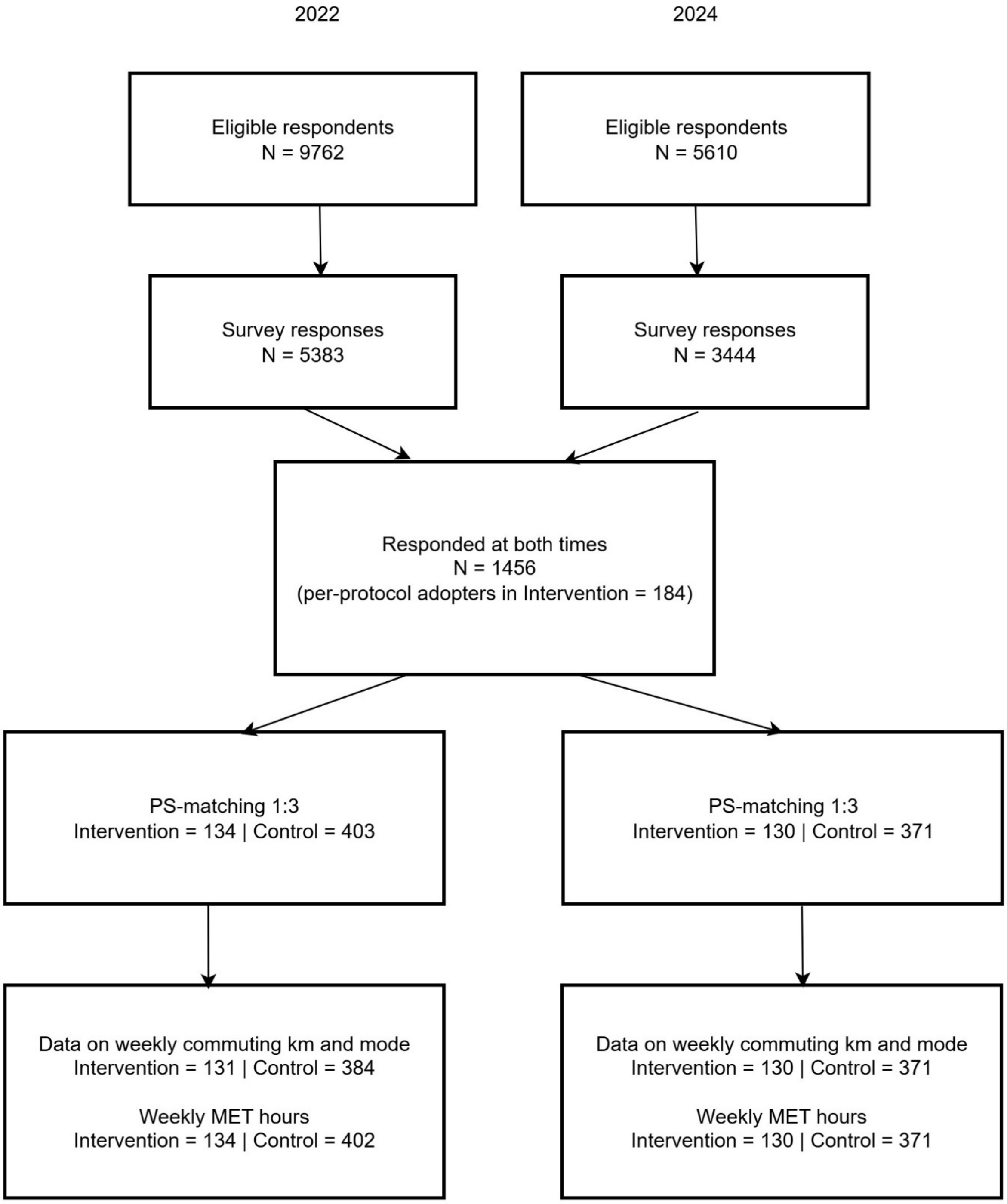

Figure S1. Flow chart of the study population in the per-protocol analysis set

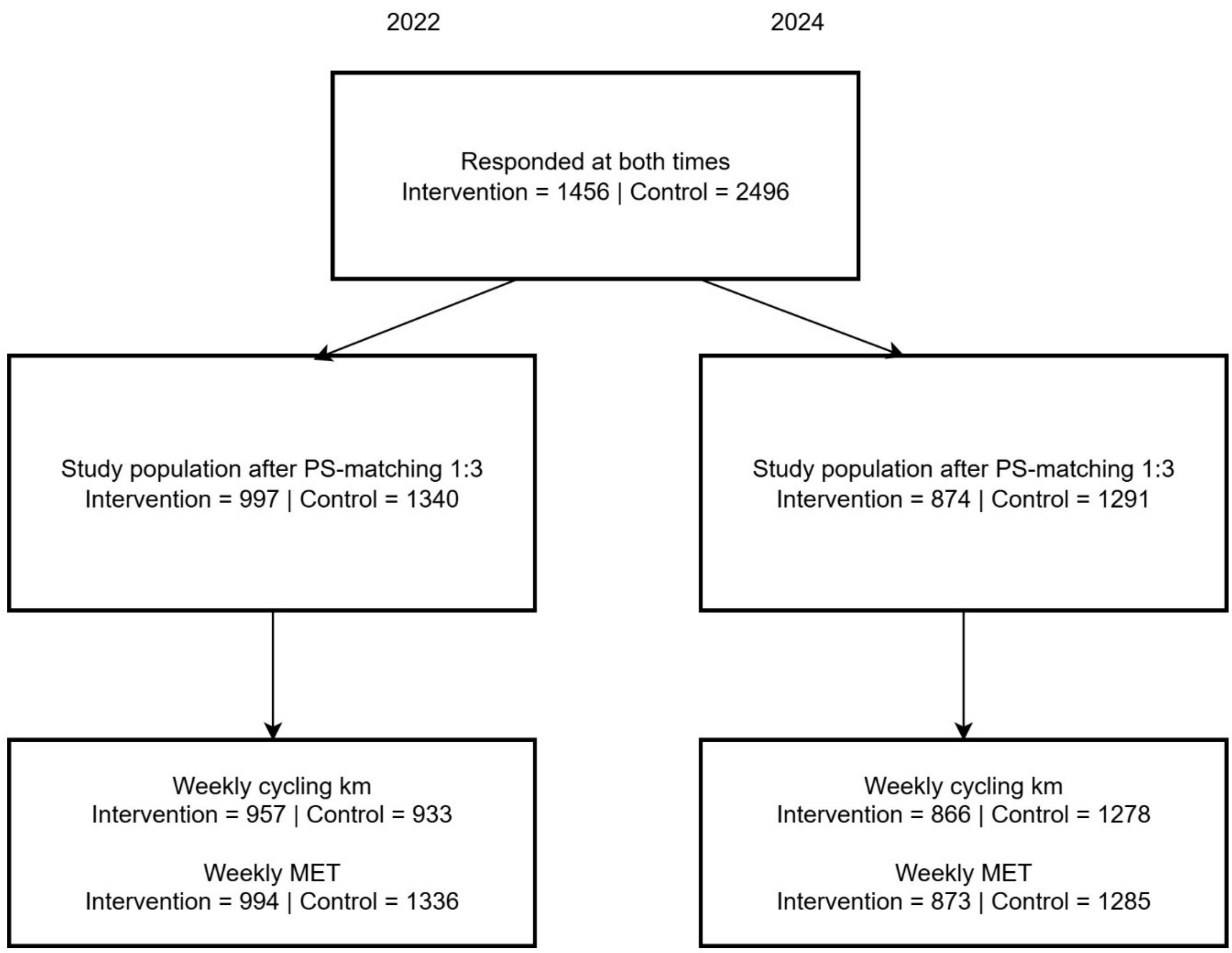

Figure S2. Flow chart of the study population in the non-adopters analysis set

Table S3. PS-adjusted model-based least square means (LS-means) derived from repeated-measures generalized estimating equations (GEE) Poisson models (log link), back-transformed from the log scale; error bars show robust 95% confidence intervals.

Analysis set	Outcome	Group	Year	LS-mean	95% CI lower	95% CI upper
**Intention-to-treat**	**Bicycle commute Summer (km/week)**	Control	2022	15.3	13.5	17.4
		Control	2024	14.3	12.3	16.6
		Intervention	2022	14.4	13	16
		Intervention	2024	16.8	15.2	18.5
	**Bicycle commute Winter (km/week)**	Control	2022	4.9	3.8	6.5
		Control	2024	4.7	3.3	6.8
		Intervention	2022	5.5	4.6	6.6
		Intervention	2024	6	5	7.1
	**Commuting by car Summer (km/week)**	Control	2022	79.9	74.1	86.1
		Control	2024	80.1	74.8	85.8
		Intervention	2022	70.8	64.9	77.3
		Intervention	2024	63.1	57.5	69.2
	**Overall physical activity (MET hours/week)**	Control	2022	0.9	0.8	1
		Control	2024	0.9	0.8	1
		Intervention	2022	1	0.9	1.1
		Intervention	2024	1	0.9	1.2
**Per protocol**	**Bicycle commute Summer (km/week)**	Control	2022	15	12.7	17.8
		Control	2024	15.6	13.1	18.4

		Intervention	2022	22.4	18.4	27.4
		Intervention	2024	36.8	31	43.6
	**Bicycle commute Winter (km/week)**	Control	2022	5	3.6	7
		Control	2024	6.1	4.3	8.6
		Intervention	2022	9.8	7	13.7
		Intervention	2024	15.3	11.7	20
	**Commuting by car Summer (km/week)**	Control	2022	66.8	57.2	78
		Control	2024	64.8	54.8	76.6
		Intervention	2022	78.3	63.1	97.1
		Intervention	2024	62.6	49.5	79.3
	**Overall physical activity (MET hours/week)**	Control	2022	40.4	37.4	43.5
		Control	2024	42.3	39.2	45.6
		Intervention	2022	41.3	36.7	46.5
		Intervention	2024	44.5	39.4	50.2
**Non-adopters**	**Bicycle commute Summer (km/week)**	Control	2022	15	13.2	17.2
		Control	2024	15	12.9	17.3
		Intervention	2022	13.5	12.1	15.1
		Intervention	2024	14	12.5	15.7
	**Bicycle commute Winter (km/week)**	Control	2022	4.8	3.6	6.5
		Control	2024	5.1	3.6	7.2
		Intervention	2022	4.9	4	6
		Intervention	2024	5.1	4.1	6.2
	**Overall physical activity (MET hours/week)**	Control	2022	39.4	38	41
		Control	2024	40.1	38.5	41.6

		Intervention	2022	39.2	37.4	41
		Intervention	2024	39.1	37.3	41.1

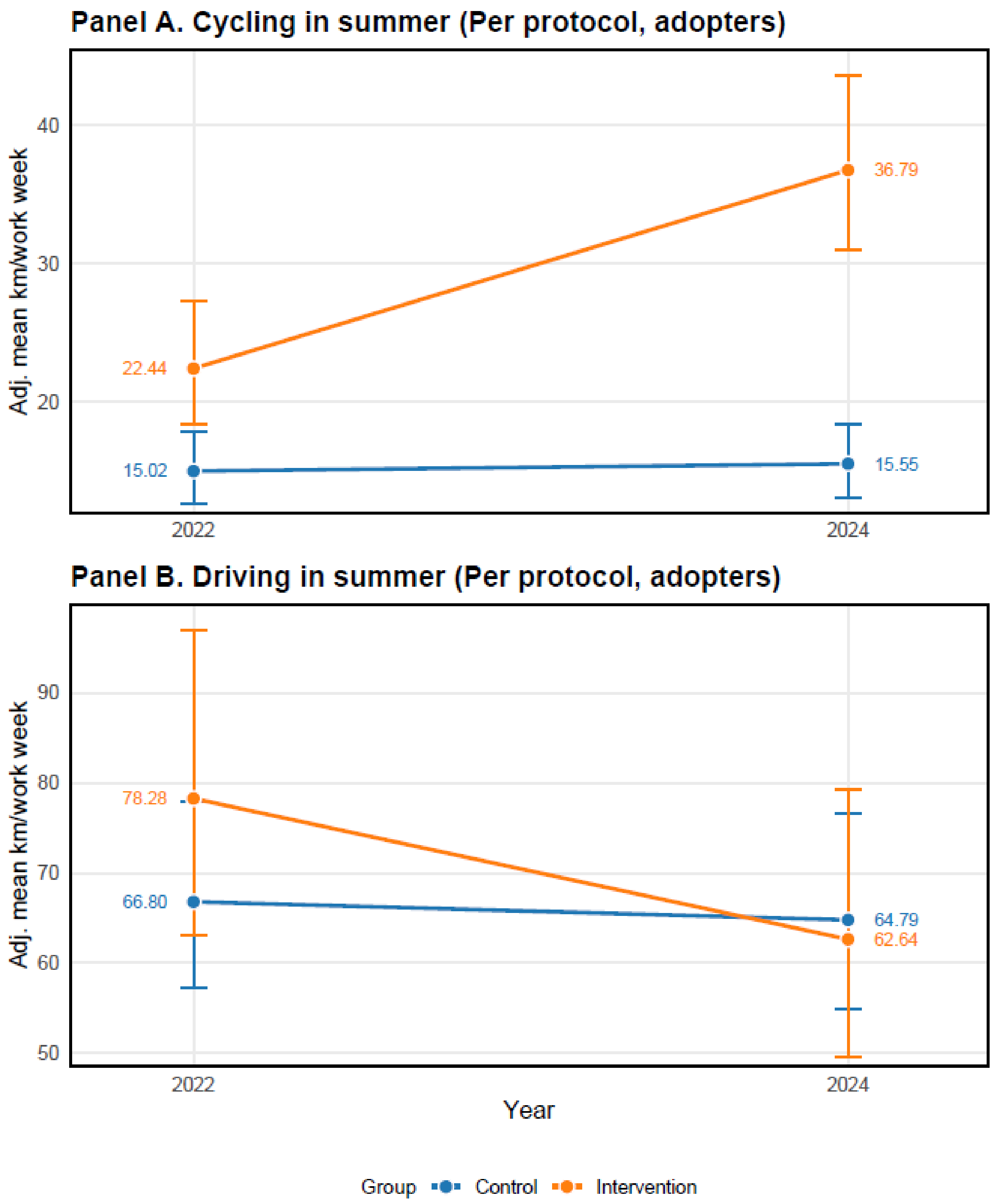

Figure S3. The adjusted means of weekly kilometers commuted by bicycle per protocol (bicycle benefit adopters). Panel A: Cycling in summer weather. Panel B: Car commuting in

summer weather. Error bars indicate 95% confidence intervals.

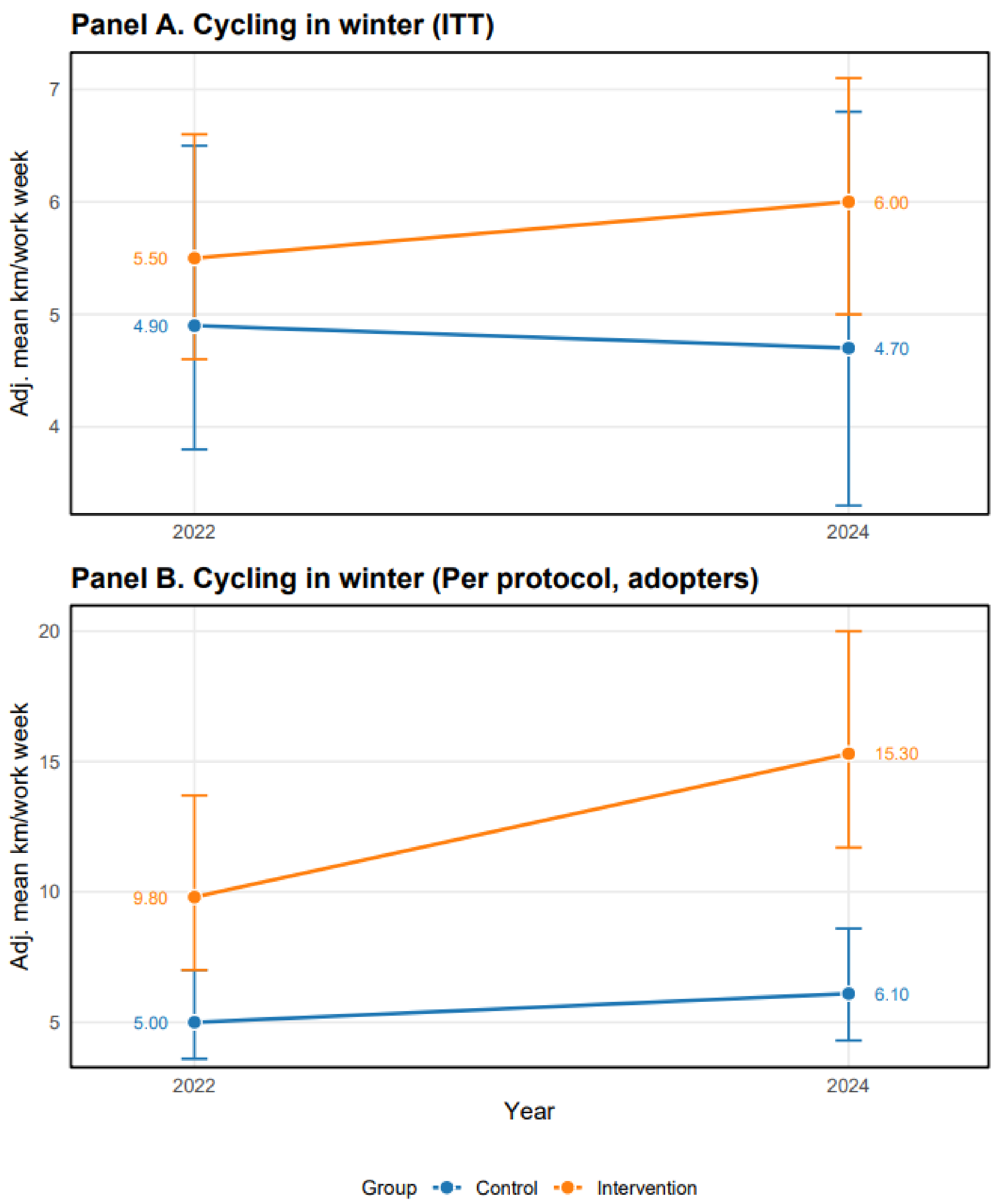

Figure S4. The adjusted means of weekly kilometers commuted by bicycle in ITT and per protocol (bicycle benefit adopters). Panel A: Bicycle commute in winter weather (ITT). Panel B: Bicycle commuting in winter weather (Per protocol/adopters). Error bars indicate 95% confidence intervals.

Supplement 2. Detailed description of study measures

Outcomes

The respondents were asked the following question about their commuting habits: "How often do you commute in the summer weather by (1) walking; (2) cycling; (3) public transport with 1000 m or more of walking or cycling; (4) public transport with less than 1000 m of walking or cycling; (5) private car use, either as a driver or a passenger?" The same question was repeated for winter weather. The response options were: 1 = daily or almost daily; 2 = a few times a week; 3 = once a week; 4 = less than once a week; 5 = never. (1–3) Participants were also asked to report their one-way commute distance in kilometers (km) at each time point. If the participant reported using at least one commute mode in summer and one in winter weather, the commuting behavior data was considered complete, and the missing values were imputed with 'never' (4). As the intervention encouraged cycling, commuting by bicycle was chosen as the primary outcome variable, and car use was used as secondary outcome. The reported frequency was converted to represent the average workdays per week: 'daily or almost daily' = 5 days; 'a few times a week' = 3 days; 'once a week' = 1 day; 'less than once a week' = 0.5 days; 'never' = 0 days. To produce the average weekly kilometers of commute by bicycle and car, we multiplied the number of weekly commuting days by travel mode by the round-trip distance of the daily commuting (2, 5). This was done separately for both summer and winter weather.

Overall physical activity was measured using questions about the average weekly hours of physical activity or exercise during leisure time and commuting within the previous 12 months, with varying intensity corresponding to walking, brisk walking, jogging, and running. The response categories were: < 30 minutes, 1 hour, 2–3 hours, and > 4 hours. We

used the following scales for calculations: <30 minutes = 15 minutes, 1 hour = 45 minutes, 2–3 hours = 2.5 hours, and >4 hours = 5 hours. The time spent on each activity at each intensity level, in hours per week, was multiplied by the average energy expenditure of that activity and expressed in metabolic equivalent task (MET) hours. Physical activities evaluated to correspond to walking, brisk walking, jogging, and running, were given MET values 3.5, 5, 8, and 11 MET, respectively (6).

Covariates

We collected information on the respondents' age (continuous variable), sex (male or female), and socio-economic status (SES) from the employers' registers. Based on 2001 International Standard Classification of Occupations (ISCO) codes, SES was categorized into three groups; high (managers and senior specialists), intermediate (associate professionals and clerical/service workers), and low (manual workers), with typical examples including physicians and teachers (high), office and customer-service personnel and health/social care workers (intermediate), and construction, cleaning, and practical nurses (low). We also had survey-based information on the type of job contract (temporary/permanent), job tenure (years with employer and years in the current position), working time (full or part-time; day or shift work; years in shift work), remote work (part-time/full-time), and marital status (having a partner; yes/no), children aged 0-6 years (yes/no), children aged 7-18 years (yes/no), and individual commute distance (continuous variable; one-way commute distance in kilometres).

Alcohol use was defined through questions on weekly consumption. One drink was approximately equivalent to one unit, or one glass of alcoholic beverage, or 12 g of alcohol. Alcohol use was dichotomized into no use or moderate use (max of 140 g or 11 units per

week for women and 280 g or 23 units per week for men) versus alcohol use greater than this (7). Smoking was dichotomized into current smokers and non-smokers (including never and ex-smokers) (8).

The assessment of job strain was based on the Job Demands and Control model (9) and was conducted using a modified Job Content Questionnaire (10). Responses were given on a Likert scale, from 1 = “very little” to 5 = “very much”. The continuous measure for job strain (balance between job demands and job control; JD/JC) was constructed by dividing the mean response scores on psychological job demands (5 items) by the mean response scores on job control (9 items), so that a higher value indicated higher strain (11).

Recovery from work was measured using a single item: ‘Let's assume that 10 points indicate that you fully recover from work before the next workday, and 0 points that you do not recover at all. How would you score your recovery from work?’ The item is based on a measure developed and used by Kinnunen et al. (12, 13), and it has been shown to correlate with potential antecedents and consequences of poor recovery. In this study, instead of the original scale of 1–5, we used a scale from 0 (=not at all) to 10 (=completely). The measure was treated as a continuous variable (14).

Variables used in subgroup analysis

Perceived health is a single item “How do you rate your health?” with response options from 1=poor to 5=good (15). Self-rated work ability is a single-item measure from the Work Ability Index (16, 17): “Let’s assume that your work ability at its all-time best would be given 10 points, and 0 points would indicate that you are completely unable to work. How would you score your current work ability?” (18). Self-reported body mass index (weight in

kg divided by height in m) is dichotomized as less than 30 (non-overweight) and 30 or more (overweight) (19).

Emission calculation

To illustrate the potential climate benefits of reduced car commuting, we estimated annual changes in tailpipe CO_2-equivalent (CO_2e) emissions for the intervention municipality using within-group change from the ITT analysis. The calculation was based on the observed decrease in weekly car commuting distance during the summer months, to account for reduced cycling during winter (–7.7 km per working week between 2022 and 2024). We assumed 46 working weeks (20) per year and a four-month winter period with no measurable effect, corresponding to 30.7 "summer" working weeks per year. Annual reductions in kilometers driven per employee were multiplied by the 2024 national emission factor for passenger-car travel provided by VTT (110 g CO_2e per km, equivalent to 0.110 kg/km)(21). Total avoided emissions were then obtained by multiplying the per-employee reduction by the number of eligible employees in the intervention municipality in 2024 (N = 5610). For contextual interpretation, the aggregate reduction was compared with average annual emissions from a typical passenger car (12 000 km × 110 g/km = 1.32 t CO_2e per vehicle per year).

1. Kalliolahti E, Aalto V, Salo P, Lanki T, Ervasti J, Oksanen T. Associations between commute mode use and self-rated health and work ability among Finnish public sector employees. Scand J Public Health. 2023 Mar 20;140349482311592. doi:10.1177/14034948231159212

2. Haukka E, Gluschkoff K, Kalliolahti E, Lanki T, Jussila JJ, Halonen JI, et al. Changes in active commuting and changes in health: Within- and between-individual analyses among 16 881 Finnish public sector employees. Prev Med. 2023;177:107744–107744. doi:10.1016/j.ypmed.2023.107744

3. Makkonen A, Gluschkoff K, Airaksinen J, Halonen JI, Salo P, Ervasti J. Development of a multifactorial prediction model for commute mode choice in 10 983 Finnish public sector employees: a cross-sectional study. BMJ Open. 2024;14(10):e080276-. doi:10.1136/bmjopen-2023-080276

4. Kalliolahti E, Gluschkoff K, Haukka E, Lanki T, Jussila JJ, Halonen JI, et al. Changes in active commuting and changes in work ability and recovery from work in 16,778 Finnish public sector employees. J Transp Health. 2024;38:101872-. doi:10.1016/j.jth.2024.101872

5. Kalliolahti E, Gluschkoff K, Lanki T, Halonen JI, Salo P, Oksanen T, et al. Associations Between Active Commuting and Sickness Absence in Finnish Public Sector Cohort of 28 485 Employees. Scand J Med Sci Sports. 2024;34(12):e70001-n/a. doi:10.1111/sms.70001

6. Ervasti J, Airaksinen J, Pentti J, Vahtera J, Suominen S, Virtanen M, et al. Does increasing physical activity reduce the excess risk of work disability among overweight individuals? Scand J Work Environ Health. 2019;45(4):376–85. doi:10.5271/sjweh.3799

7. Ervasti J, Kivimäki M, Head J, Goldberg M, Airagnes G, Pentti J, et al. Sickness absence diagnoses among abstainers, low-risk drinkers and at-risk drinkers: consideration of the U-shaped association between alcohol use and sickness absence in four cohort studies. Addict Abingdon Engl. 2018;113(9):1633–42. doi:10.1111/add.14249

8. Heikkilä K, Nyberg ST, Fransson EI, Alfredsson L, De Bacquer D, Bjorner JB, et al. Job strain and tobacco smoking: an individual-participant data meta-analysis of 166,130 adults in 15 European studies. PloS One. 2012;7(7):e35463–e35463. doi:10.1371/journal.pone.0035463

9. Karasek, R, Theorell, T. Healthy work: stress, productivity, and the reconstruction of working life. Appl Ergon. 1992;23(5):352–352. doi:10.1016/0003-6870(92)90320-U

10. Karasek R, Brisson C, Kawakami N, Houtman I, Bongers P, Amick B. The Job Content Questionnaire (JCQ): An Instrument for Internationally Comparative Assessments of Psychosocial Job Characteristics. J Occup Health Psychol. 1998;3(4):322–55. doi:10.1037/1076-8998.3.4.322

11. Ruokangas SM, Weiste E, Ervasti J, Oksanen T, Nieminen P. Job demands and job control among occupational therapists in public sector in Finland. Scand J Occup Ther. 2022 Jan 2;29(1):69–78. doi:10.1080/11038128.2020.1849396 PubMed PMID: 33242265.

12. Kinnunen U, Feldt T, Siltaloppi M, Sonnentag S. Job demands-resources model in the context of recovery: Testing recovery experiences as mediators. Eur J Work Organ Psychol. 2011;20(6):805–32. doi:10.1080/1359432X.2010.524411

13. Kinnunen U, Feldt T. Job Characteristics, Recovery Experiences and Occupational Well-being: Testing Cross-lagged Relationships across 1 Year. Stress Health. 2013;29(5):369–82. doi:10.1002/smi.2483

14. Selander K, Korkiakangas E, Toivanen M, Yli-Kaitala K, Kangas H, Nevanperä N, et al. Engaging Leadership and Psychological Safety as Moderators of the Relationship between Strain and Work Recovery: A Cross-Sectional Study of HSS Employees. Healthc Basel. 2023;11(7):1045-. doi:10.3390/healthcare11071045

15. Robine J, Jagger C. Creating a Coherent Set of Indicators to Monitor Health Across Europe: The Euroreves 2 Project. The Gerontologist. 2002;42(1):35–35.

16. Ilmarinen J, Tuomi K, Klockars M. Changes in the work ability of active employees over an 11-year period. Scand J Work Environ Health. 1997;23:49–57.

17. Tuomi K, Ilmarinen J, Martikainen R, Aalto L, Klockars M. Aging, work, life-style and work ability among Finnish municipal workers in 1981—1992. Scand J Work Environ Health. 1997;23:58–65.

18. Ahlstrom L, Grimby-Ekman A, Hagberg M, Dellve L. The Work Ability Index and single-item Question: Associations with Sick Leave, Symptoms, and health-a Prospective Study of Women on long-term Sick Leave. Scand J Work Environ & Health. 2010;36(5):404–12. doi:10.5271/sjweh.2917

19. Halonen JI, Kivimäki M, Pentti J, Stenholm S, Kawachi I, Subramanian SV, et al. Green and blue areas as predictors of overweight and obesity in an 8-year follow-up study. Obes Silver Spring Md. 2014;22(8):1910–7. doi:10.1002/oby.20772

20. Kuntatyönantajat. IV luku Vuosiloma [Chapter IV Annual Leave] [Internet]. 2025 [cited 2025 Sep 15]. Available from: https://www.kt.fi/sopimukset/kvtes/2022-2025/vuosiloma

21. Markkanen, Johanna; Lauhkonen, Arttu; Niemi, Anni. Scenarios for greenhouse gas emissions and energy consumption of road transport in Finland: Exploring the impact of existing policies [Internet]. VTT Technical Research Centre of Finland; 2023. Report VTT Technology No. 413. Available from: https://publications.vtt.fi/pdf/technology/2023/T413.pdf
